# Radiotherapy was associated with the lower incidence of metachronous second primary lung cancer

**DOI:** 10.1038/s41598-019-55538-4

**Published:** 2019-12-17

**Authors:** Zhi Gang Hu, Yu Feng Tian, Wen Xin Li, Fan Jun Zeng

**Affiliations:** 1 Respiratory Disease Research Institute of China, The First College of Clinical Medical Science, Three Gorges University, NO. 183 Yiling Road, Yichang, 443003 People’s Republic of China; 2 Department of Respiratory Medicine, Yichang Central People’s Hospital, NO. 183 Yiling Road, Yichang, 443003 People’s Republic of China; 3 Department of Respiratory Medicine, Renmin Hospital of Wuhan University, Wuhan, Hubei Province People’s Republic of China; 4 Yichang Central People’s Hospital, Three Gorges University, NO. 183 Yiling Road, Yichang, 443003 People’s Republic of China

**Keywords:** Cancer prevention, Lung cancer

## Abstract

Our study aims to estimate the incidence of metachronous second primary lung cancer(SPLC) in initial primary lung cancer(IPLC) survivors and to determine whether radiotherapy affects the risk of metachronous SPLC in the first five years after the diagnosis of lung cancer. Incidence data of IPLC individuals who survived ≥2 years were obtained from SEER-18 database in 2004–2007. Joinpoint regression analysis and competing risk analysis were used to calculate the incidence of metachronous SPLC. Propensity score matching and decision analysis were available to estimate the effect of radiotherapy on metachronous SPLC. 264 of 11657 IPLC survivors with radiotherapy and 1090 of 24499 IPLC survivors without radiotherapy developed metachronous SPLC during 5-year follow-up, respectively. In joinpoint regression analysis, the 5-year incidence of metachronous SPLC in the radiotherapy group was lower than that in the nonradiotherapy group(2385 per 100,000 vs 4748 per 100,000, HR = 0.43,95% CI:0.39–0.47). Competing risk analysis showed that the survivors with radiotherapy were associated with the lower 5 year incidence of metachronous SPLC compared with those without radiotherapy(2.28% vs 4.47%, HR = 0.49,95% CI:0.43–0.57). Through propensity score matching, 4077 pairs of survivors were available to further study that radiotherapy potentially decreased the risk of developing metachronous SPLC with the adjustment of various factors(2.5% vs 3.3%, HR = 0.72, 95% CI:0.55–0.96). Decision analysis suggested that radiotherapy was a negative independent risk factor of metachronous SPLC with clinical net benefit in a range of risk thresholds (2% to 5%). Survivors of IPLC with radiotherapy likely had a low risk of metachronous SPLC during the first five years follow-up, especially non-small cell lung cancer.

## Introduction

Radiotherapy is an important treatment option for lung cancer regardless of cancer forms, such as non small cell lung cancer or small cell lung cancer. In comparison with chemotherapy or surgery alone, radiotherapy combined with chemotherapy or surgery can effectively prolong the survival time of some patients of lung cancer^1–3^. For lung cancer patients with contraindications of chemotherapy and surgery, radiotherapy also was considered as the initial treatment option. Unfortunately, radiation exposure caused by radiotherapy may bring about some adverse events, mainly including radiotherapy-related pneumonitis and cardiac toxicity^4^. In addition, excessive radiation exposure likely increases the risk of cancer. Radiation exposure secondary to low dose computed tomography for lung cancer screening was found to potentially increase the lifetime attributable risk of lung cancer and major cancers, especially in women aged 50–54^5^. The previous studies about radiotherapy and the risk of developing lung cancer in the breast cancer patients were inconsistent^6–10^. A recent system review declared that older radiotherapy techniques for the treatment of breast cancer seemingly increased the risk of developing lung cancer in the ipsilateral lung, while there was no explicit evidence of an increased risk with modern techniques^11^. However, It is noteworthy that the latency period of most radiation-induced cancers is at least 5 years to 10 years^6–8^. The clinical benefit of radiotherapy in terms of cancer treatment should be verified in the early period after the diagnosis of cancer.

Some previous epidemiological studies reported that the incidence of lung cancer for initial primary lung cancer(IPLC) individuals was approximately 1% to 2% per year, which was fourfold to sixfold higher than those with no history of lung cancer^12–14^. Compared with normal population, IPLC individuals theoretically have more genetic predisposition and higher exposure to carcinogenic agents. Therefore, IPLC individuals can be classified as a high-risk population of lung cancer.The present results about the association between radiotherapy and the risk of second primary lung cancer(SPLC) were controversial. Tucker and his colleagues^15^ claimed that the risk of developing SPLC of the patients with chest radiotherapy was approximately two fold higher than those without chest radiotherapy in small cell lung cancer. Another study from Khanal *et al*.^16^ declared that patients exposed to radiotherapy harbored a lower excess risk of SPLC when compared to patients without radiotherapy in lung adenocarcinoma and squamous cell carcinoma. Han *et al*.^17^ found that age, histology, and extent of IPLC were the important factors in predicting SPLC risk, while radiotherapy had no significant impact on SPLC risk.

In this study, our first aim is to estimate the incidence of developing metachronous SPLC in the individuals with IPLC who received radiotherapy through joinpoint regression analysis and competing risk analysis. The second aim of our study is to determine whether radiotherapy is associated with the incidence of metachronous SPLC in the first five years, which was performed through propensity score matching and decision analysis. According to the accepted diagnostic criterion of Martini and Melamed^18^, the patients who were diagnosed as metachronous SPLC need to fulfill the following situations: (1)IPLC and SPLC are reported different histology; (2) When IPLC and SPLC display same histology, the following criteria must be met:(1)the disease-free interval between IPLC and SPLC must be more than 2 years. (2) IPLC and SPLC origin from carcinoma *in situ*, or occur in different lobes with no metastatic carcinoma of common lymph nodes and no extrapulmonary metastasis at the time of diagnosis.

## Methods

### Data source and collection

Surveillance, Epidemiology, and End Results (SEER) database of the National Cancer Institute is a population-based database with the strictest data-quality indicators and consistent criteria. We obtained the detained data of the IPLC individuals who survived ≥2 years between 2004 and 2007, thus performed a five years follow-up for IPLC survivors, until the diagnosis of a new primary cancer, until death, or the end of follow-up. Histological classification was performed in accordance with the International Classification of Diseases of Oncology, 3rd edition. We focused on this period for two reasons: On the one hand, the deadline of present SEER data was Dec 2014 and provided additional treatment information. On the other hand, the stage of lung cancer was based on the American Joint Committee on Cancer sixth Edition staging system since 2004. Lung cancer was roughly divided into five categories, including adenocarcinoma, squamous cell carcinoma, large cell carcinoma, small cell carcinoma, and other. The IPLC individual was not considered when a patient’s histological characteristics were unclear and could not be classified into the aforementioned five categories, such as malignant neoplasm (8000/3), malignant tumor cell(8001/3), carcinoma(8010/3), etc. All participants of this study must include positive diagnostic confirmation, explicitly diagnostic time(year and month), and survival time. The participant who had a histology of lung cancer before 2004 was excluded. The information provided in the radiotherapy record and the radiotherapy sequence with surgery were used as a basis for determining whether the individuals with IPLC received radiotherapy. We also excluded the individuals whose radiotherapy information were unknown. Furthermore, we also collected the following demographic variables: Age, Sex, Race, Marital status at diagnosis, Histology, Tumor size, CS lymph nodes, CS distant metastasis, Stage group of IPLC, Tumor stage, Chemotherapy record, and survival time.

### Ethics

Because all data derived from open SEER database, there were no patients involved in the recruitment and conduct of the study. This study was deemed exempt for review by the Institutional Review Board at China, Three Gorges University.

### Statistical analysis

#### The incidence of metachronous SPLC

Surveillance research program of American National Cancer Institute recommends the application of joinpoint regression analysis to estimate cancer incidence per 100,000 and cancer incidence trend with adjustment of the potential confounding effect of age^19^. Based on the Monte Carlo permutation method, joinpoint analysis can be performed to describe continuous changes of cancer incidence and determine the number of joinpoints with the slope in trends as well as their corresponding significance. In joinpoint regression analysis, Monte Carlo permutation methods provide P value of each joinpoint, and Bonferroni correction maintains the overall asymptotic significance level. In addition, joinpoint analysis provides Annual Percentage Change(APC) using age-specific Poisson regression models with a log-link function^19^. Standard population was considered as the patients who were diagnosed between 2004 and 2007, who also had positive histology and accurate year and month of diagnosis. In recent years, competing risk analysis was widely used to estimate the incidence and trend of cancer. Because the individuals with IPLC may die before developing SPLC, standard Cox regression potentially leads to a substantial bias in risk estimation of SPLC^17^. Competing risk analysis can provide a cumulative incidence function to evaluate the unbiased risks of metachronous SPLC through the application of proportional subdistribution hazards regression^18,20^. To better assess the incidence of metachronous SPLC, we performed these two kinds of analyses and compared the difference between joinpoint regression analysis and competing risk analysis.

#### The impact of radiotherapy on the incidence of metachronous SPLC

Competing risk analysis was used to find the potential risk factors of developing metachronous SPLC and to estimate the interactions of all possible risk factors through stepwise forward and backward elimination methods. Akaike information criterion and the Bayesian information criterion (BIC) were used to competing risks models^17^. Propensity score matching analysis could better evaluate the effect of radiotherapy on the incidence of metachronous SPLC through the adjustment of various factors^21^. As the last component in the evaluation of clinical usefulness of variable, decision analysis provided visual graphical curve to estimate the net benefit against the threshold probability of the variable^22^. In this study, decision analysis might reveal whether radiotherapy was the independent risk factor of developing metachronous SPLC with net benefit in a threshold probability.

Joinpoint software, Stata software, SPSS software and R software were used to complete the above-mentioned analyses. R package ‘cmprsk’ was adopted to complete competing risk analysis. Decision analysis was performed through R package ‘rmda’. When *P* values were less than 0.05, a two-sided statistical significance was considered.

## Results

### Study design and patients’ outcome

**Figure 1:**
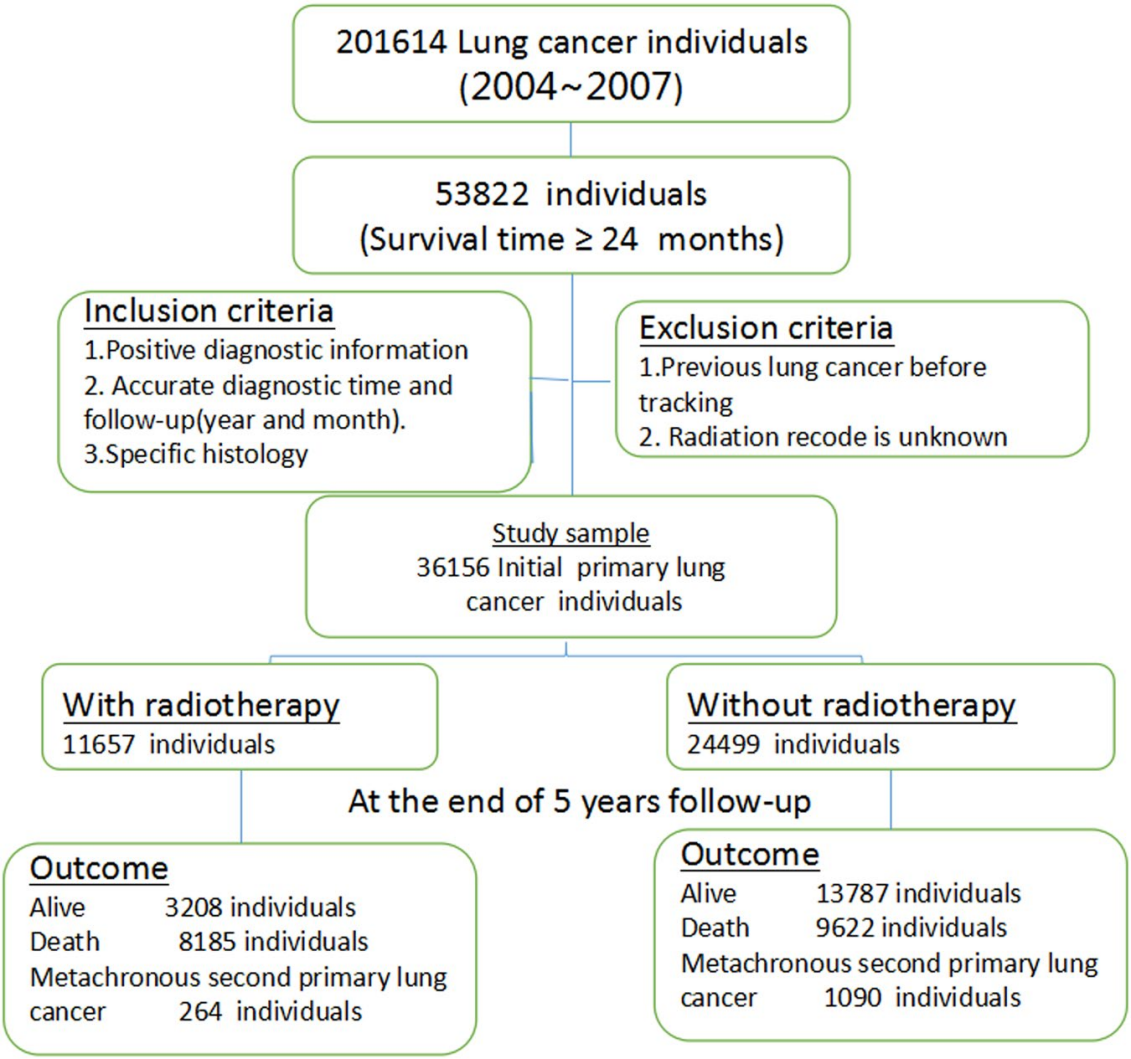
An algorithm of the study design and patient selection from Surveillance, Epidemiology, and End Results (SEER) database of the National Cancer Institute.

A total of 201614 individuals were diagnosed lung cancer between 2004 and 2007 in SEER 18 registries. Approximately 26.7% of lung cancer, including 53822 individuals, survived more than two years. According to our inclusion criteria and exclusion criteria, 36156 IPLC individuals were incorporated in this study, including 11657 individuals with radiotherapy and 24499 individuals without radiotherapy respectively. After 5 year follow-up, there were 264(2.3%) individuals who developed metachronous SPLC, 8185(70.2%) individuals who died and 3208(27.5%) individuals who survived in the radiotherapy group, respectively. In the control group, there were 1090(4.45%) individuals who developed metachronous SPLC, 9622(39.3%) individuals who died and 13787(56.3%) individuals who survived, respectively. The study algorithm was presented in Fig. 1.

### Clinical characteristics of initial primary lung cancer

**Figure 2:**
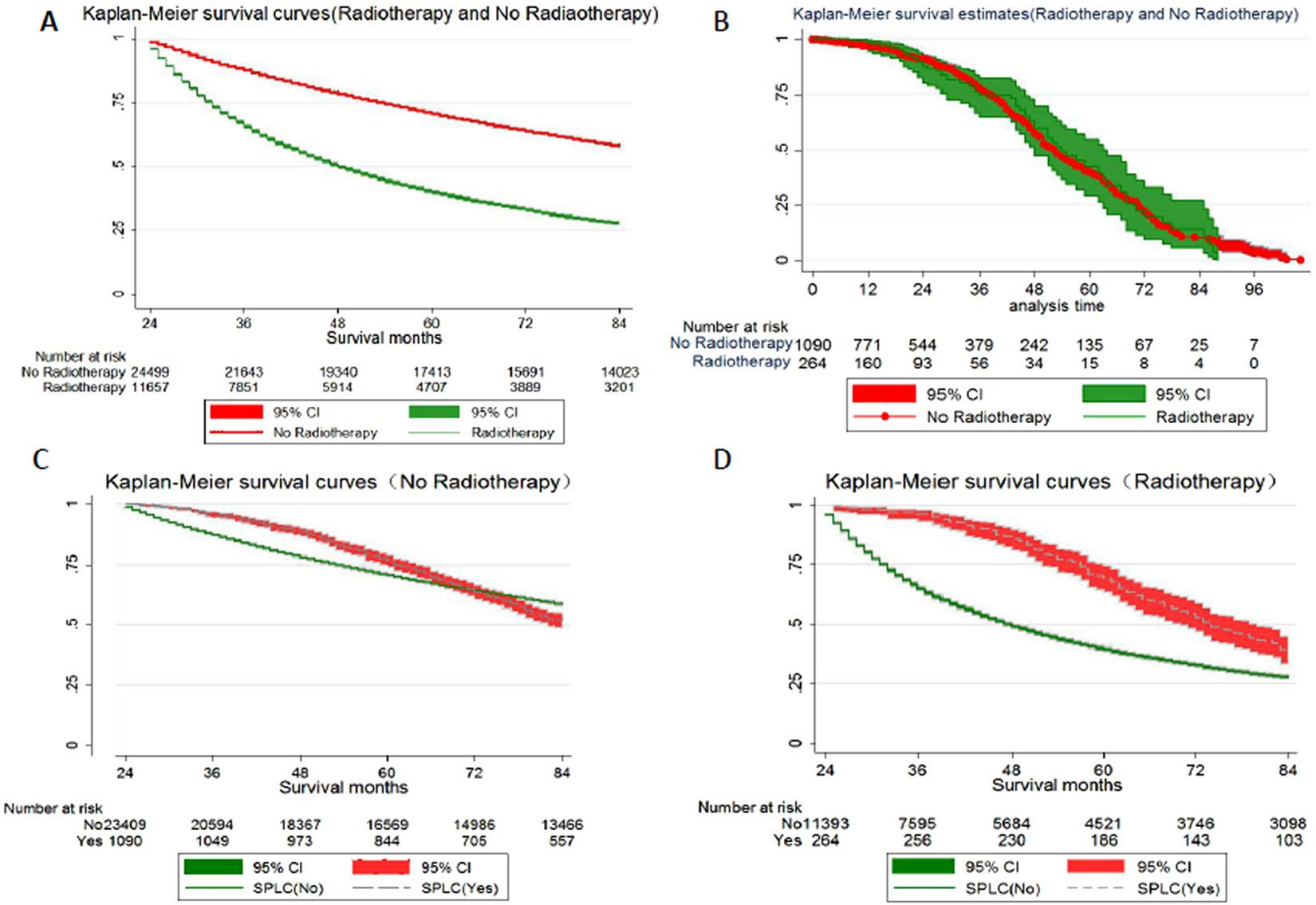
Kaplan-Meier curve of Overall Survival in different groups: (**A**) The survival time in no radiotherapy group was significantly longer(HR = 0.33, 95% CI: 0.32–0.35, *P* < 0.001) than that in radiotherapy group. (**B**) After the occurrence of metachronous SPLC, survival times among two groups were similar(HR = 1.0, 95% CI: 0.76–1.33, *P* = 0.96, (**C**) The estimated survival time was longer among patients with metachronous SPLC than among patients without metachronous SPLC in radiotherapy group(HR = 1.54, 95% CI:1.36–1.74). (**D**) No significant difference of survival time between patients with metachronous SPLC and patients without metachronous SPLC was found in no radiotherapy group(HR = 0.91, 95% CI:0.83–1.00).

Table 1 showed the distribution of demographic, clinical, treatment and survival time stratified by radiotherapy or no radiotherapy. Female individuals were more than men in both two groups. Patients aged 70 to 79 years in the nonradiotherapy group were more compared with those in the radiotherapy group(33.9% vs 26.8%). The individuals were distributed equally among the age groups from 0 to 49 years, from 60 to 69 years, and 80 years or older. The number of patients with tumour sizes of less than 3.5 cm in the nonradiotherapy group was more than that in the radiotherapy group(70.6% vs 30.8%). In histological aspect, there were less adenocarcinoma (41.3% vs 58.7%) and more small cell carcinoma(19.9% vs 0.7%) in the radiotherapy group than those in the nonradiotherapy group. In terms of tumor stage, early stage patients(stage I and stage II) and advanced stage patients(stage III and stage IV) in the nonradiotherapy group respectively were 78.3% and 13%, while early stage patients and advanced stage patients in the radiotherapy group respectively were 26.4% and 76.4%. In terms of treatment, the proportion of patients who received chemotherapy in the radiotherapy group was significantly higher than that in the nonradiotherapy group(77.5% vs 19.3%). Figure 2 showed survival curves for patients among different groups. On account of more early stage patients in the nonradiotherapy group, their survival time was significantly longer(HR = 0.33, 95% CI: 0.32–0.35, *P* < 0.001) than that in the radiotherapy group(see Fig. 2A). After the occurrence of SPLC, survival times among two groups were similar(HR = 1.0, 95% CI: 0.76–1.33, P = 0.96, see Fig. 2B). The development of metachronous SPLC seemingly did not significantly decrease the overall survival of the patients, whether in the radiotherapy group(see Fig. 2C) or in the nonradiotherapy group(see Fig. 2D). The estimated survival time was longer among patients with metachronous SPLC than that among patients without metachronous SPLC in the radiotherapy group(HR = 1.54, 95% CI:1.36–1.74, see Fig. 2C). No significant difference of survival time between patients with metachronous SPLC and patients without metachronous SPLC was found in the nonradiotherapy group (HR = 0.91, 95% CI:0.83–1.00, see Fig. 2D).

**Table 1:** Baseline characteristics of included.

	No Radiotherapy	Radiotherapy	P-value
**N**	24499	11657	
**Sex**			<0.001
Female	13437 (54.8%)	5909 (50.7%)	
Male	11062 (45.2%)	5748 (49.3%)	
**Age**	66.8 ± 11	66.8 ± 10.9	0.45
**Race**			<0.001
Black	1775 (7.2%)	1283 (11.0%)	
White	21274 (86.8%)	9608 (82.4%)	
Other	1415 (5.8%)	753 (6.5%)	
Unknown	35 (0.1%)	13 (0.1%)	
**Marital status**			<0.001
Married	14673 (59.9%)	6697 (57.5%)	
Unmarried	9211 (37.6%)	4654 (39.9%)	
Unknown	615 (2.5%)	306 (2.6%)	
**Tumor size**			<0.001
<3.5 cm	17294 (70.6%)	4295 (36.8%)	
3.5 to 6.9 cm	5727 (23.4%)	3944 (33.8%)	
≥7.0 cm	800 (3.3%)	1220 (10.5%)	
Unknown	678 (2.8%)	2198 (18.9%)	
**Histology**			<0.001
Adenocarcinoma	14388 (58.7%)	4814 (41.3%)	
Squamous carcinoma	6032 (24.6%)	3489 (29.9%)	
Large cell carcinoma	776 (3.2%)	492 (4.2%)	
Small cell carcinoma	176 (0.7%)	2325 (19.9%)	
Other	3127 (12.8%)	537 (4.6%)	
**CS lymph nodes**			<0.001
N0	20138 (82.2%)	4013 (34.4%)	
N1/N2/N3	4131 (16.8%)	7105 (61%)	
Unknown	230 (0.9%)	539 (4.6%)	
**CS Metastasis**			<0.001
No	23315 (95.2%)	8889 (76.3%)	
Yes	893 (3.6%)	2407 (20.6%)	
Unknown	291 (1.2%)	361 (3.1%)	
**Stage group**			<0.001
I	16765 (68.4%)	2040 (17.5%)	
II	2431 (9.9%)	1032 (8.9%)	
III	2352 (9.6%)	5466 (46.9%)	
IV	831 (3.4%)	2395 (20.5%)	
Unknown	2120 (8.7%)	724 (6.2%)	
**Chemotherapy**			<0.001
No/unknown	19762 (80.7%)	2618 (22.5%)	
Yes	4737 (19.3%)	9039 (77.5%)	
**Median survival months**	69.6	54	<0.001
**Outcome**			<0.001
Alive	13787 (56.3%)	3208 (27.5%)	
Metachronous SPLC	1090 (4.4%)	264 (2.3%)	
Death	9622 (39.3%)	8185 (70.2%)	

Abbreviations: CS, Condensed stage; IPLC, Initial primary lung cancer; SPLC,

Second primary lung cancer.

### Clinical characteristics of metachronous second primary lung cancer

A total of 1354 IPLC individuals developed metachronous SPLC during 5 years follow-up, with 712 females and 642 males. The detailed histological distributions of IPLC with metachronous SPLC were shown in Table S1. In the radiotherapy and non-radiotherapy group, adenocarcinoma and squamous cell carcinoma were the main histology of metachronous SPLC, followed by small cell carcinoma, other histology and large cell carcinoma. The mean interval time between IPLC and metachronous SPLC was 52.7 ± 17.07 months in radiotherapy group, which was similar to that in no radiotherapy group(51.7 ± 17.13 months, *P* = 0.45).

### The incidence of metachronous SPLC

During 5 years follow-up, the cumulative incidence of metachronous SPLC was 2.3%(260/11657) in the radiotherapy group, which was lower than that in the nonradiotherapy group(4.45%, 1090/24499). In joinpoint analysis, age-adjusted 1 and 5 years cumulative incidence of metachronous SPLC respectively was 617 per 100000, and 2385 per 100000 in the radiotherapy group. By comparison, the risk of developing metachronous SPLC in the nonradiotherapy group seemed to be higher with 1448 per 100000 of age-adjusted 1 year cumulative incidence and 4748 per 100000 of age-adjusted 5 year cumulative incidence. When considering the competing risk of all cause death, the patients with radiotherapy still were associated with lower 5 year incidence of metachronous SPLC compared with those without radiotherapy (2.28% vs 4.47%, HR = 0.49, 95% CI:0.43–0.57). The 1 year incidence of metachronous SPLC in the radiotherapy group and the nonradiotherapy group respectively was 0.5% and 1.08%, respectively. Overall, the risk of developing metachronous SPLC seemingly was higher in patients without radiotherapy than patients with radiotherapy (see Table 2).

**Table 2:** Cumulative risk of metachronous second primary lung cancer.

Year	Joinpoint regression analysis(per 100,000)					Competing risk analysis(%)		
	Radiotherapy	APC	No Radiotherapy	APC	HR	Radiotherapy	No Radiotherapy	HR
1	616.81		1448.42			0.5	1.08	
2	864.93	34.4	1948.96	40.2	0.43	1.03	2.06	0.4
3	1212.87	(14.8,57.7)	2622.46	(15.9,69.6)	(0.39,0.47)	1.43	2.98	(0.43–0.57)
4	1700.78		3528.71			1.88	3.75	
5	2384.95		4748.14			2.28	4.47	

Note: APC, Annual Percentage Change.

### The impact of radiotherapy on the incidence of metachronous SPLC

**Figure 3:**
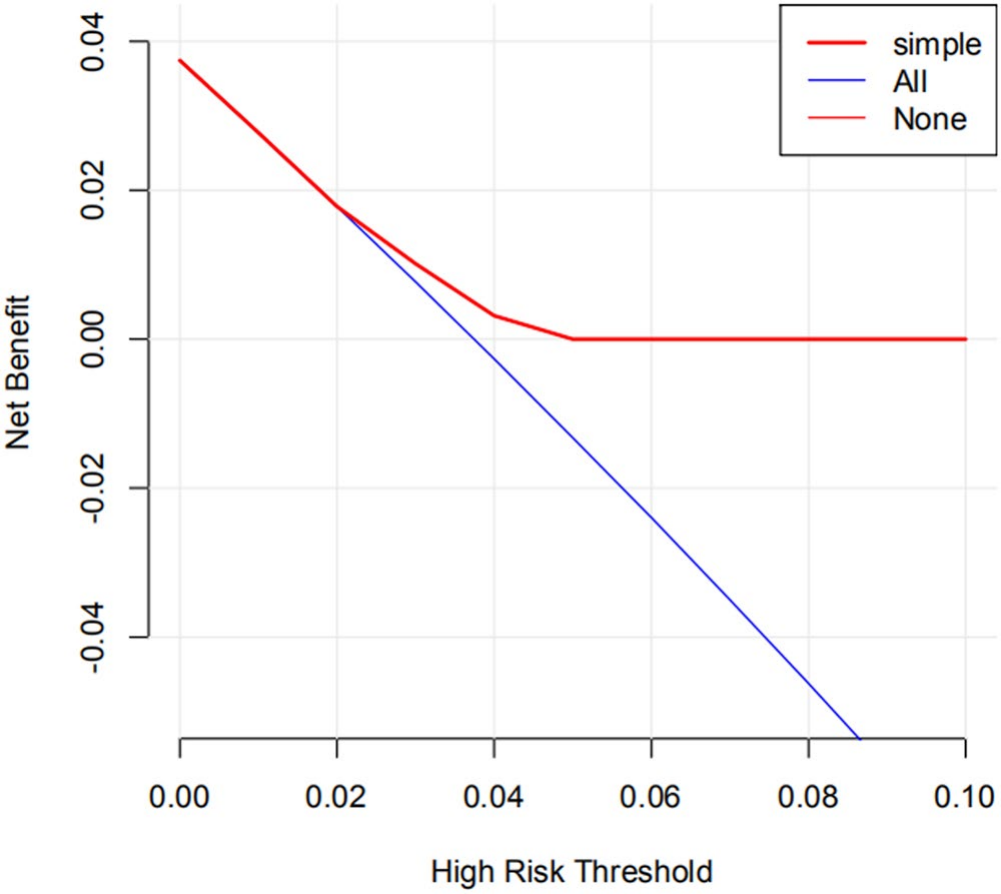
Decision analysis indicated that radiotherapy was a negatively independent risk factor of developing metachronous SPLC with clinical net benefit in a range of risk thresholds (2% to 5%).

Through competing risk analysis of single factor and various factors, two potential risk factors of SPLC, namely, stage group and histology, were found in patients with IPLC in the radiotherapy group. With regard to the stage group, the patients with IPLC at stage I were associated with the highest risk of SPLC among the stage groups.(see Table 3). In the histological aspect, the IPLC patients with small cell carcinoma had the largest risk of developing metachronous SPLC, followed by other histology, squamous cell carcinoma, large cell carcinoma, and adenocarcinoma(see Table 3). Subsequently, propensity score matching analysis was used to adjust the differences in the abovementioned factors between the radiotherapy group and the nonradiotherapy group. A total of 4077 pairs of IPLC patients were available to evaluate the impact of radiotherapy on the incidence of metachronous SPLC(see Table 4). The 5 year cumulative incidence of metachronous SPLC in the radiotherapy group was less than that in the nonradiotherapy group(2.5% vs 3.3%, HR = 0.72, 95% CI:0.55–0.95). Decision analysis indicated that radiotherapy was a negative independent risk factor of developing metachronous SPLC with clinical net benefit in a range of risk thresholds (2% to 5%, see Fig. 3).

**Table 3:** Risk factors associated with metachronous second primary lung cancer in radiotherapy group.

	1-year	2-year	3-year	4-year	5-year	Unvariate analysis	*P*	Multivariate analysis	*P*
**Stage group**									
I	0.98%	1.72%	2.40%	3.24%	3.83%	—		—	
II	0.29%	0.97%	1.26%	1.65%	1.95%	0.49 (0.29–0.84)	0.01	0.49 (0.29–0.85)	0.01
III	0.48%	1.04%	1.47%	1.93%	2.41%	0.60 (0.44–0.81)	0.00	0.58 (0.41–0.82)	0.00
IV	0.21%	0.42%	0.59%	0.72%	0.81%	0.23 (0.13–0.38)	<0.01	0.25 (0.14–0.45)	<0.01
Unknown	0.55%	1.11%	1.39%	1.81%	2.23%	0.56 (0.31–1.02)	0.06	0.60 (0.32–1.13)	0.11
**Histology**									
Adenocarcinoma	0.37%	0.67%	1.02%	1.30%	1.55%	—		—	
Squamous carcinoma	0.52%	1.12%	1.64%	2.19%	2.62%	1.68 (1.21–2.35)	0.00	1.35 (0.95–1.92)	0.09
Large cell carcinoma	0.41%	0.61%	1.43%	1.84%	2.46%	1.40 (0.72–2.74)	0.33	1.33 (0.62–2.84)	0.46
Small cell carcinoma	0.73%	1.64%	1.90%	2.50%	3.12%	1.98 (1.39–2.81)	0.00	1.87 (1.27–2.76)	0.00
Other	0.56%	1.49%	1.68%	2.44%	2.82%	1.85 (1.02–3.34)	0.04	1.61 (0.86–3.02)	0.14

**Table 4:** Baseline characteristics after propensity score matching analysis.

	No Radiotherapy	Radiotherapy	P-value
**N**	4077	4077	
**Age**	67.1 ± 10.1	67.2 ± 11.5	0.692
**Sex**			0.163
Female	2051 (50.3%)	2114 (51.9%)	
Male	2026 (49.7%)	1963 (48.1%)	
**Race**			0.56
Black	403 (9.9%)	400 (9.8%)	
White	3420 (83.9%)	3399 (83.4%)	
Other	254 (6.2%)	278 (6.8%)	
**Marital status**			0.224
Married	2374 (58.2%)	2428 (59.6%)	
Unmarried	1703 (41.8%)	1649 (40.4%)	
**Laterality**			0.486
Left	1675 (41.1%)	1706 (41.8%)	
Right	2402 (58.9%)	2371 (58.2%)	
**Histology**			0.738
Squamous cell carcinoma	2381 (58.4%)	2387 (58.5%)	
Adenocarcinoma	1281 (31.4%)	1269 (31.1%)	
Small cell carcinoma	192 (4.7%)	192 (4.7%)	
Large cell carcinoma	73 (1.8%)	89 (2.2%)	
Other	150 (3.7%)	140 (3.4%)	
**Tumor size**	35.9 ± 20.8	35.5 ± 19.4	0.392
**CS lymph nodes**			0.608
N0	2257 (55.4%)	2280 (55.9%)	
N1/N2/N3	1820 (44.6%)	1797 (44.1%)	
**Distance metatasis**			0.492
M0	3608 (88.5%)	3588 (88.0%)	
M1	469 (11.5%)	489 (12.0%)	
**Stage group**			0.363
I	1584 (38.9%)	1544 (37.9%)	
II	657 (16.1%)	708 (17.4%)	
III	1367 (33.5%)	1336 (32.8%)	
IV	469 (11.5%)	489 (12.0%)	
**Chemotherapy**			0.158
No/Unknown	1708 (41.9%)	1771 (43.4%)	
Yes	2369 (58.1%)	2306 (56.6%)	
**Survival times**	58.6 ± 23.1	58.9 ± 22.7	0.613
**Outcomes**			0.028
Alive	1455 (35.7%)	1396 (34.2%)	
Death	2487 (61.0%)	2578 (63.2%)	
SPLC	135 (3.3%)	103 (2.5%)	

Abbreviations: SPLC = Second primary lung cancer.

## Discussion

In our study, the age-adjusted incidence of developing metachronous SPLC was 0.62% at 1 year and 2.39% at 5 years among the IPLC survivors with radiotherapy. When considering the competing risk of all cause death, the cumulative incidence of developing metachronous SPLC was 0.5% at 1 year and 2.28% at 5 years among IPLC survivors with radiotherapy. The IPLC survivors without radiotherapy seemed to have a higher risk of metachronous SPLC than those with radiotherapy, which also were identified in propensity score matching and decision analysis. In addition, we also found that the presence of metachronous SPLC did not reduce overall survival of IPLC patients, either in the radiotherapy group or in the nonradiotherapy group. Our study demonstrated that the 5-year incidence of small cell lung cancer in radiotherapy group was similar to that in non-radiotherapy group(3.1% vs 2.8%, sbHR = 0.87, 95% CI: 0.33–2.07). However, radiotherapy for initial primary non small cell lung cancer seemingly decreased the 5-year incidence of metachronous SPLC compared with that in the nonradiotherapy group, mainly including adenocarcinoma, large cell carcinoma, and squamous cell carcinoma. To the best of our knowledge, this study was the largest one that focused on the incidence of metachronous SPLC after IPLC with radiotherapy through joinpoint regression analysis and competing risk analysis. In addition, our study suggested that the occurrence of metachronous SPLC did not significantly shorten the overall survival of patients with IPLC.

Most studies have reported that the rate of developing SPLC was 1% to 2% per patient per year^12–14^. In 2001, Jeremic and colleagues^23^ reported that the incidence of developing metachronous SPLC in long-term survivors of early(I/II) non small cell lung cancer after radiotherapy alone was 1.0% (95% CI: 0.1–1.9%) per year during the first 5-year period, which was slightly higher than our study. One possible explanation is that the study population in the study of Jeremic *et al*. focused on early(I/II) lung cancer patients with radiotherapy alone. The present studies demonstrated that early lung cancer patients harbored higher risk of metachronous SPLC compared with advanced lung cancer patients because the longer survival time after successful treatment of lung cancer gave such patients more chance for development of metachronous SPLC^24,25^. Our study also confirmed that the IPLC patients IPLC at stage I with 0.98% per year were associated with a highest risk of developing metachronous SPLC than those in the advanced stage groups. This observation was similar to the result obtained by Jeremic and colleagues^23^. However, the occurrence rate of SPLC may increase during the first and second 5-year periods^23,26,27^. Therefore, the surveillance of SPLC should be a constant and long-term process.

All the time, radiation exposure during radiotherapy is considered a potential risk factor of lung cancer. Early studies have reported that the relative risks for metachronous SPLC of small-cell lung cancer patients with radiotherapy increased approximately twofold than patients without radiotherapy^6,15^. However, our study suggested that the risk of developing metachronous SPLC for IPLC patients with radiotherapy was lower compared with that of patients without radiotherapy in the first 5 years follow-up period, which was consistent with the study of Abdel-Rahman *et al*.^25^. Even when we adjusted various factors of metachronous SPLC through propensity score matching analysis, this decreased risk of metachronous SPLC in the radiotherapy group still remained. A recent study from Abdel-Rahman and his colleagues^25^ assessed the risk of subsequent primary thoracic cancer among IPLC patients in 9 SEER registries, treated between 1988 and 2013. Relative risk for the development of metachronous SPLC in the nonradiotherapy group was 1.83 (95% CI: 1.65–2.03) than in the radiotherapy group during 1 to 5 years follow-up since lung cancer diagnosis. However, no significant difference in the incidence of metachronous SPLC between the radiotherapy group and the nonradiotherapy group was found at both 5–10 years (relative risk 0.95 [95% CI:0.85–1.07]) and 10 or more years of follow-up (relative risk 0.98 [95% CI 0.82–1.16]). Khanal and colleagues^16^ reported on 12,246 patient with stage Ia non-small cell lung cancer registered with SEER-13 between January 2004 and December 2010. The excess risk of metachronous SPLC was significantly lower in the radiotherapy group than in the nonradiotherapy group after approximately 5 years median follow-up. Another study from Han *et al*.^17^ evaluated the risk of developing metachronous SPLC in 20,032 long-term survivors (>5 years) of IPLC between 1988 and 2003. There was no significant difference in the risk of developing metachronous SPLC between radiotherapy group and non-radiotherapy group after 8 years median follow-up. On the basis of these studies, we found that the risk of SPLC among patients with IPLC subjected to radiotherapy was lower than that among patients with IPLC not subjected to radiotherapy in the first 5 year follow-up period, while the difference among two groups would become inconspicuous in the subsequent follow-up period. Our decision analysis also suggested that radiotherapy could obtain clinical net benefit in a range of risk thresholds (2% to 5%). When the cumulative risk of metachronous SPLC gradually increases with the extension of follow-up and exceeds 5%, clinical net benefit should disappear. The benefit of SPLC caused by radiotherapy may be significantly greater than the risk of SPLC secondary to radiotherapy, which was more obvious in the first 5 year follow-up period.

There were the following strengths in our study. Firstly, our study population came from SEER-18 database, including 36156 IPLC individuals and covering approximately 28% of the U.S. population^28^. Large sample multicenter studies could potentially decrease the selection and referral bias of single-center study and small-sample study. Meanwhile, the strictest data-quality indicators and the consistent criteria for collecting data in all SEER registries can ensure the quality of its variables. Secondly, the authors estimated the incidence of metachronous SPLC in IPLC patients with radiotherapy at 1 year and 5 years through joinpoint regression analysis and competing risk analysis. By comparison, competing risk analysis be applied to obtain unbiased risks in the presence of competing risks and produce more accurate estimates for the incidence of metachronous SPLC. Thirdly, this study was the first to simultaneously use propensity score matching and decision analysis for evaluating the effect of radiotherapy on the incidence of SPLC.

The absence of information on detailed smoking history was an important limitation for our study. Some studies indicated that small cell lung cancer patients with the history of smoking harbored a significantly increased risk of SPLC secondary to radiotherapy^14,15^. A comparison of 365 early-stage non small cell lung cancer patients with stereotactic body radiation therapy reported that the risk of developing metachronous SPLC was 1.8%(6/327) for patients with the history of smoking, and 0%(0/37) for nonsmokers (*P* < 0·05)^29^. A recent study by Kono *et al*.^24^ indicated that the number of pack-years of smoking (*P* = 0.21) and smoking status(*P* = 1) do not affect the incidence of metachronous SPLC for small cell lung cancer with radiotherapy and chemotherapy. Our study showed that the patients with squamous cell carcinoma (known to be associated with heavy smoking) harbored the higher risk of metachronous SPLC compared with adenocarcinoma in non small cell lung cancer with radiotherapy(2.62% vs 1.55% sbHR = 1.68, 95% CI:1.21–2.53, *P* < 0.001). SEER-18 database did not include the detailed information about smoking and other potentially important factors of metachronous SPLC, like any exposure to carcinogen and family history of cancer. The lack of these data possibly influenced the estimate about the association between radiotherapy and metachronous SPLC. Another important limitation was that SEER database did not report specific details with regard to the recipient of anatomic site radiation. A few stage IV patients maybe only receive radiotherapy for bone or brain metastases, which potentially affected the results. Although we evaluated the impact of radiotherapy on metachronous SPLC through multiple methods, our retrospective study inevitably harbored some potentially selective biases. Although the authors have utilized Martini and Melamed criteria to define SPLC, we should also understand that some of these malignancies are either recurrent or metastatic disease of IPLC.

For long term survivors who underwent radiotherapy after the diagnosis of IPLC, the incidence of metachronous SPLC gradually increased over time with approximately 0.5% to 0.6% per year. The risk of SPLC was likely higher in the nonradiotherapy group than in the radiotherapy group during the first 5 year follow up period. With the development of modern techniques, the risk of developing lung cancer secondary to radiotherapy is likely to decrease. Radiotherapy possibly provides clinical benefits to the treatment of IPLC and the prevention of SPLC. However, these conclusions are yet to be verified by conducting further studies.

## Supplementary information

Dataset 1

## Data Availability

The data underlying this study were obtained from the National Cancer Institute’s Surveillance, Epidemiology, and End Results Program(SEER ID: huzg). All relevant data are within the paper and its Supporting Information Files.
