# Supplementary material for: Radiotherapy was associated with the lower incidence of metachronous second primary lung cancer
Source: Sci Rep. 2019 Dec 17;9:19283. doi: 10.1038/s41598-019-55538-4 (PMC6917769; doi:10.1038/s41598-019-55538-4)
Supplement: Supplementary file 1 — Dataset 1 [file 41598_2019_55538_MOESM1_ESM.doc]

Radiotherapy was associated with the lower incidence of metachronous second primary lung cancer.

Zhi Gang Hu ^1,2,3 &*^, Yu Feng Tian^4&^, Wen Xin Li ^1,2^, Fan Jun Zeng ^1,2*^

**Authors’ affiliations:**

1. Respiratory Disease Research Institute of China, The First College of Clinical Medical Science, Three Gorges University, NO. 183 Yiling Road, Yichang 443003, People’s Republic of China.

2. Department of Respiratory Medicine, Yichang Central People's Hospital, NO. 183 Yiling Road, Yichang 443003, People’s Republic of China.

3. Department of Respiratory Medicine, Renmin Hospital of Wuhan University, Wuhan, Hubei Province, People’s Republic of China

4. Yichang Central People's Hospital, Three Gorges University, NO. 183 Yiling Road, Yichang 443003, People’s Republic of China.

**Running Head :** radiotherapy and metachronous second primary lung cancer

**Keyword:** Lung cancer, second primary, radiotherapy

& Zhi Gang Hu and Yu Feng Tian contribute equally to this work. They are listed as co-first author.

**Corresponding author:** Zhi Gang Hu, Department of Respiratory medicine, Respiratory Disease Research Institute of China, The First College of Clinical Medical Science, Three Gorges University, NO. 183 Yiling Road, Yichang 443003, People’s Republic of China. E-mail: [hxq910813@163.com](mailto:hzg7602589@126.com)

**Conflict of interest:** The authors declare they have no competing interests.

**Funding:** No funding.

**Text Word:** 3662  **Table:** 3 Figure: 2

| Table S1: Histological distributions of IPLC and SPLC | | | | | | | | | | |
| --- | --- | --- | --- | --- | --- | --- | --- | --- | --- | --- |
| Radiotherapy | | | |  | | No Radiotherapy | | | |  |
| IPLC |  | SPLC |  | |  | IPLC |  | SPLC |  |  |
| Histology | N | Histology | N | |  | Histology | N | Histology | N |  |
| SQ | 91 | SQ | 27 | |  | SQ | 291 | SQ | 134 |  |
|  |  | AD | 36 | |  |  |  | AD | 82 |  |
|  |  | LC | 1 | |  |  |  | LC | 6 |  |
|  |  | SC | 23 | |  |  |  | SC | 51 |  |
|  |  | Other | 4 | |  |  |  | Other | 18 |  |
| AD | 74 | SQ | 19 | |  | AD | 668 | SQ | 105 |  |
|  |  | AD | 36 | |  |  |  | AD | 489 |  |
|  |  | LC | 1 | |  |  |  | LC | 9 |  |
|  |  | SC | 11 | |  |  |  | SC | 31 |  |
|  |  | Other | 7 | |  |  |  | Other | 34 |  |
| LC | 12 | SQ | 2 | |  | LC | 41 | SQ | 14 |  |
|  |  | AD | 7 | |  |  |  | AD | 17 |  |
|  |  | SC | 3 | |  |  |  | LC | 2 |  |
| SC | 72 | SQ | 36 | |  |  |  | SC | 6 |  |
|  |  | AD | 27 | |  |  |  | Other | 2 |  |
|  |  | LC | 4 | |  | SC | 5 | SQ | 1 |  |
|  |  | SC | 2 | |  |  |  | AD | 4 |  |
|  |  | Other | 3 | |  | Other | 85 | SQ | 15 |  |
| Other | 15 | SQ | 3 | |  |  |  | AD | 43 |  |
|  |  | AD | 8 | |  |  |  | LC | 2 |  |
|  |  | LC | 1 | |  |  |  | SC | 8 |  |
|  |  | SC | 2 | |  |  |  | Other | 17 |  |
|  |  | Other | 1 | |  |  |  |  |  |  |
| **Abbreviations:** AD, adenocarcinoma; IPLC, Initial primary lung cancer; LC, Large cell; SC, Small-cell lung cancer; SPLC, Second primary lung cancer; SQ,Squamous cell. | | | | | | | | | | |
